# d‐Sorbose Promotes Regulatory T‐Cell Differentiation and Ameliorates Autoimmune and Allergic Diseases via Hexosamine Biosynthetic Pathway Inhibition

**DOI:** 10.1096/fj.202601110R

**Published:** 2026-08-28

**Authors:** Fumiaki Sato, Masato Hoshi, Hiroyuki Yokoi, Sayaka Yoshida, Ryosuke Takenaka, Nao Shimoga, Nanaka Morita, Chieko Tashita, Kyoka Yamazaki, Yuka Kishimoto, Hiroyuki Tezuka, Hiroyasu Ito, Kuniaki Saito

**Affiliations:** ^1^ Department of Disease Systems Analysis Medicine Fujita Health University School of Medical Sciences Toyoake Aichi Japan; ^2^ Department of Medical Science Technology, School of Health Science at Narita International University of Health and Welfare Narita Chiba Japan; ^3^ Department of Medical Technology Gifu University of Medical Science Seki Gifu Japan; ^4^ Research and Development Matsutani Chemical Industry Co. Ltd. Itami Hyogo Japan; ^5^ Department of Cellular Function Analysis, Research Promotion Headquarters Fujita Health University Toyoake Aichi Japan; ^6^ Department of Joint Research Laboratory of Clinical Medicine Fujita Health University School of Medicine Toyoake Aichi Japan; ^7^ Department of Advanced Diagnostic System Development Fujita Health University School of Health Sciences Toyoake Aichi Japan

**Keywords:** d‐sorbose, hexosamine biosynthetic pathway, non‐obese diabetes (NOD) model, OVA‐induced asthma model, rare sugar, regulatory T cell

## Abstract

Monosaccharides are fundamental biomolecules involved in various biological processes. Rare sugars, naturally scarce monosaccharide derivatives, exhibit unique physiological effects independent of standard energy metabolism. Among them, d‐sorbose, the C‐3 epimer of d‐fructose, has poorly understood biological functions. This study investigated the immunometabolic effects of d‐sorbose, focusing on regulatory T‐cell (Treg) differentiation and immune modulation. Ex vivo assays using splenic naïve CD4^+^ T cells revealed that d‐sorbose significantly increased Foxp3^+^ Treg frequencies to levels comparable to d‐mannose, without inducing the CD8^+^ T cell reduction observed with d‐mannose. In vivo, continuous d‐sorbose administration ameliorated ovalbumin‐induced airway inflammation and prevented autoimmune diabetes in non‐obese diabetic mice by selectively increasing interleukin‐10‐producing Tregs in regional lymph nodes and inflamed tissues. Histological analyses revealed reduced inflammatory cell infiltration and preserved tissue architecture in d‐sorbose‐treated groups. Metabolomic profiling indicated that d‐sorbose suppressed glycolysis, the pentose phosphate pathway, and the hexosamine biosynthetic pathway (HBP), as evidenced by decreased UDP‐GlcNAc levels, while elevating the tricarboxylic acid cycle intermediate malate. Pharmacological inhibition of glutamine–fructose‐6‐phosphate aminotransferase, combined with glutamine restriction and GlcNAc rescue assays, demonstrated that d‐sorbose promotes Treg differentiation through coordinated metabolic reprogramming of glucose partitioning and glutamine catabolism rather than passive HBP inhibition. Notably, d‐sorbose treatment did not alter T‐cell survival and demonstrated a favorable safety profile during long‐term administration. These findings identify d‐sorbose as a novel immunoregulatory sugar that promotes functional Treg differentiation by orchestrating intracellular glucose and glutamine metabolism, highlighting its potential as a dietary or therapeutic agent for controlling inflammatory and autoimmune diseases.

## Graphical abstract

d‐Sorbose promotes Foxp3^+^ Treg differentiation ex vivo and exerts potent anti‐inflammatory effects in vivo, ameliorating allergic airway inflammation and preventing autoimmune diabetes without compromising T cell viability or inducing toxicity. Mechanistically, we show that d‐sorbose promotes Treg differentiation through coordinated metabolic reprogramming by altering glucose partitioning, suppressing glycolysis, the PPP, and the HBP, and modulating glutamine metabolism.

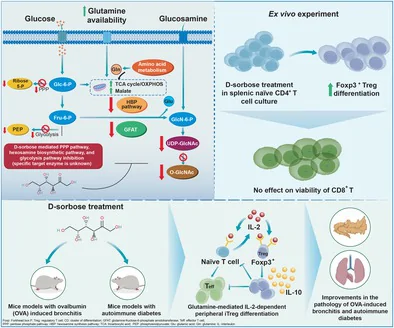

## 1 Introduction

Monosaccharides, the smallest functional units of carbohydrates, exist in nature both as individual molecules and as components of various biomolecules, such as glycans, glycoproteins, and nucleic acids, and play fundamental roles in biological processes. In contrast, rare sugars are monosaccharides and their derivatives that are scarce in nature. Although approximately 50 types of rare sugars have been identified, they account for only 0.1% of all naturally occurring sugars. These rare sugars generally have a sweet taste but are not readily utilized as energy sources. Recently, rare sugars have been reported to exert various physiological effects [1, 2]. For example, d‐allulose alleviates Type II diabetes [3] and inhibits cancer cell proliferation [4] by activating autophagy [5]. Among these rare sugars, d‐sorbose, a 3, 4‐epimer of d‐fructose, is synthesized from d‐tagatose using d‐tagatose 3‐epimerase and d‐allulose‐3‐epimerase [6, 7]. d‐Sorbose, which has approximately 70% of the sweetness of sucrose, is transported via glucose transporter (GLUT) 5 but not sodium–glucose cotransporter 1 in the small intestine of rats [8]. In humans and rats, 25% of orally ingested d‐sorbose is excreted in urine after intestinal absorption, 24% is fermented and utilized by intestinal bacteria, and 51% is excreted in feces [8, 9]. However, the regulation of intracellular glucose (Glu) metabolism pathways by d‐sorbose or its effects on lymphocyte differentiation remains largely unexplored.

In humans and mice, d‐mannose stimulates regulatory T cell (Treg) differentiation by promoting transforming growth factor‐beta (TGF‐β) activation in vitro [10]. Furthermore, d‐mannose alleviates autoimmune disease symptoms by inducing Tregs, which suppress dendritic cell maturation in a cytotoxic T‐lymphocyte‐associated protein 4‐dependent manner [11]. Upon stimulation by antigen‐presenting cells (APCs), naïve T cells differentiate into effector T cells or Tregs. This differentiation fate depends on cellular energy metabolism [12, 13]. Effector T cells (Teffs) exhibit highly activated glycolytic systems and lipid synthesis pathways, whereas Tregs exhibit mixed metabolic profiles that rely on glycolysis and on lipolysis for energy production [13]. Indeed, deletion of GLUT1 in CD4^+^ T cells inhibits Teff differentiation and promotes Treg differentiation [14], suggesting that reprogramming plays an important role in the subset‐specific regulation of CD4^+^ T cell functions. Thus, various rare sugars may be involved in regulating T‐cell differentiation.

This study aimed to clarify the effects of the rare sugar, d‐sorbose, on T‐cell differentiation and T cell‐mediated diseases.

## 2 Materials and Methods

### 2.1 Mice

C57BL/6J male (RRID:IMSR_JAX: 000664) mice and female BALB/c mice (RRID:IMSR_ORNL:IE‐BALB‐C, MGI:2683685) were obtained from Japan Charles River (Kanagawa, Japan). NOD/ShiJcl female mice (RRID:IMSR_JCL:JCL:MDB‐0006) were obtained from CLEA Japan Inc. (Tokyo, Japan). The mice were housed in a specific pathogen‐free environment in our facility, maintained at 23°C ± 2°C under a 12‐h light/dark cycle (lights on at 8:00 AM), with free access to food and water. All experiments were performed in accordance with the Guidelines for Animal Care of Fujita Health University (approval no. AP17018) and were approved by the Animal Experimentation Committee of Fujita Health University. Procedures involving mice and their care conformed to international guidelines as described in the Principles of Laboratory Animal Care (National Institutes of Health publication 85‐23, revised 1985).

### 2.2 Reagents

Roswell Park Memorial Institute (RPMI)‐1640 medium was purchased from Fujifilm Wako Pure Chemicals (Osaka, Japan). RPMI complete medium was prepared by adding 10% fetal bovine serum (FBS) (HyClone; Cytiva, Tokyo, Japan), 100 U/mL penicillin, and 100 mg/mL streptomycin (Sigma‐Aldrich, St. Louis, MO, USA) to RPMI‐1640 medium.

The monosaccharides used in this study are listed in Table S1. d‐Glu was purchased from Nacalai Tesque (Kyoto, Japan), whereas d‐mannose, d‐allose, d‐allulose, d‐tagatose, and d‐sorbose were obtained from Matsutani Chemical Industry Co. (Hyogo, Japan). These monosaccharides were dissolved in RPMI complete medium and the concentration was adjusted to 500 mM. d‐Sorbose used for oral administration in mice was dissolved in distilled water at concentrations of 1.1 M or 0.28 M, based on a previous report [11].

The metabolites monosodium Glu‐6‐phosphate, sodium ribose‐5‐phosphate, and lactic acid were purchased from Nacalai Tesque; DL‐malic acid and monopotassium phosphoenolpyruvate from Fujifilm Wako; DL‐glyceraldehyde‐3‐phosphate from Cayman Chemical (Ann Arbor, MI, USA); and UDP‐N‐acetyl‐d‐glucosamine, disodium salt from Calbiotech (El Cajon, CA). All metabolites were dissolved in distilled water and used as standard solutions for intracellular metabolite assays.

The metabolic inhibitor KAN0438757 (KAN), 6‐aminonicotinamide (6‐AN), and 6‐diazo‐5‐oxo‐L‐norleucine (DON) were purchased from Selleck Biotech (Tokyo, Japan), Cayman Chemical, and Fujifilm Wako, respectively. The inhibitors were dissolved in dimethyl sulfoxide (DMSO; Sigma‐Aldrich) and diluted in complete RPMI medium. The final concentration of DMSO in cell cultures was adjusted to less than 0.033%.

To develop the asthma model mice, ovalbumin (OVA; A5503; Sigma‐Aldrich, St. Louis, MO, USA) was dissolved in pH 7.4 phosphate buffered saline (PBS). Aluminum hydroxide (Alhydrogel adjuvant 2%; InvivoGen, San Diego, CA, USA) was used as an adjuvant. OVA (25 μg) and aluminum hydroxide (2 mg/200 μL) were mixed.

### 2.3 Cell Isolation From Mice Tissues

Blood, tissues, and lymph nodes were harvested from mice at various time points. Splenocytes were prepared by removing the spleen capsule and passing the tissue through a 70‐μm filter. Splenocytes and blood cells were washed, and red blood cells were lysed as previously described [15].

### 2.4 Culture for Treg Differentiation

Naïve CD4^+^ T cells were isolated from the splenocytes of C57BL/6J mice (8 weeks old; male) using magnetic cell sorting (MACS; Naïve CD4^+^ T Cell Isolation Kit mouse; Miltenyi Biotec, Tokyo, Japan). Naïve CD4^+^ T cells were seeded at 1 × 10^5^ cells/well in flat‐bottom 96‐well plates and stimulated with recombinant human interleukin (IL)‐2 (50 IU/mL; Bio‐Techne, Minneapolis, MN), human recombinant TGF‐β1 (5 ng/mL; R&D Systems, Minneapolis, MN), and anti‐CD3/CD28–coated microbeads (Thermo Fisher Scientific, Waltham, MA) in the presence of 25 mM monosaccharides for 5 days at 37°C with 5% CO_2_. For the control group, RPMI complete medium was used instead of monosaccharides. Cells were analyzed by flow cytometry (Gallios; Beckman Coulter, Brea, CA).

### 2.5 Flow Cytometry Analysis

Foxp3 nuclear staining was performed using a fixation/permeabilization buffer (eBioscience; Affymetrix, Santa Clara, CA, USA). Briefly, single‐cell suspensions were incubated with anti‐CD16/32 (RRID:AB_312801, clone: 93; BioLegend, San Diego, CA, USA) to block Fc receptors and prevent nonspecific antibody (Ab) binding and with ethidium bromide (Sigma‐Aldrich) for dead cell staining. Cells were then stained with the following monoclonal antibodies (mAbs) conjugated to FITC, PE, or APC: anti‐CD3 antibody (RRID:AB_312663, clone: 17A2; BioLegend), anti‐CD4 antibody (RRID:AB_312691, clone: GK1.5; BioLegend), anti‐Foxp3 biotin antibody (RRID:AB_763540, clone: FJL‐16s; Thermo Fisher Scientific (Invitrogen), Waltham, MA, USA), and streptavidin (BioLegend). CD8^+^ cell‐surface staining was performed in a similar manner. In brief, single cells were incubated with anti‐CD16/32 (RRID:AB_312801, clone: 93; BioLegend, San Diego, CA, USA) to block Fc receptors and prevent nonspecific Ab binding, followed by staining with FITC‐conjugated anti‐CD8a antibody (RRID:AB_312745, clone: 53‐6.7; BioLegend) and 7‐AAD Viability Staining Solution (BioLegend) for dead cell staining. Stained cells were analyzed using the Galios flow cytometer (Beckman Coulter, Brea, CA, USA). Data were analyzed using Kaluza analysis software (RRID:SCR_016182, ver. 2.1; Beckman Coulter, Brea, CA, USA).

### 2.6 d‐Sorbose Safety Test

Male C57BL/6J mice (6 weeks old) were provided with a 1.1 M d‐sorbose solution as a replacement for drinking water. The control group was administered distilled water. During the treatment period, body weight, water intake, and food intake were measured every 2–3 days. Spleen and peripheral blood samples were collected 4 weeks after the initiation of treatment. Peripheral blood was centrifuged at 1400 × *g* for 10 min at 24°C, and the supernatant was collected as serum. Serum samples were stored at −80°C until biochemical analysis.

### 2.7 Biochemical Analysis

Serum levels of total protein (TP), alanine aminotransferase (ALT), creatinine (Cre), and Glu were measured using an automated biochemical analyzer (JCA‐BM9130; JEOL, Tokyo, Japan).

### 2.8 OVA‐Induced Asthma Model

The OVA‐induced asthma mouse model was developed based on previous reports [16, 17]. Balb/c mice (RRID: IMSR_ORNL:IE‐BALB‐C, MGI: 2683685) were divided into two groups and received either a 1.1 M d‐sorbose solution or distilled water ad libitum from 5 weeks of age. At 6, 7, and 8 weeks of age, 200 μL of a mixture containing OVA (25 μg) and aluminum hydroxide (2 mg) was administered intraperitoneally. Thereafter, OVA (100 μg/20 μL) was administered intranasally once daily for 1 week starting at 9 weeks of age. For Treg depletion, mice received intraperitoneal injections of anti‐mouse CD25 mAb or rat immunoglobulin (Ig) G1 isotype control Ab (clone PC‐61.5.3, 250 μg/mouse; BioXCell, West Lebanon, NH, USA) on the same day as OVA intraperitoneal administration. Blood samples and measurements of body weight, water intake, and food intake were recorded every 3 days during the administration period. Peripheral blood, lungs, and lymph node samples were collected at 10 weeks of age.

### 2.9 In Vitro Suppression Assay

CD4^+^CD25^−^T cells obtained from the lymph node of OVA‐treated mice were labeled with 1 μM carboxy fluorescein succinimidyl ester (CFSE; Invitrogen Molecular Probes, Waltham, MA, USA). CFSE‐labeled CD4^+^CD25^−^T cells (1 × 10^5^ cells) were stimulated with CD3/CD28 Dynabeads (1 μL/well; Thermo Fisher Scientific) for 3 days and co‐cultured with CD4^+^CD25^+^ Tregs at effector‐to‐target ratios of 0.1:1, 0.25:1, or 0.5:1. Cells were stained with anti‐CD3ε, and CFSE dilution in CD3^+^ cells was analyzed following 3 days of stimulation. Cell proliferation was quantified using Kaluza analysis software (RRID: SCR_016182, ver. 2.1).

### 2.10 Enzyme‐Linked Immunosorbent Assay (ELISA)

CD4^+^CD25^+^T cells or CD4^+^CD25^−^T cells isolated from the mediastinal lymph nodes of OVA‐treated mice using MACS magnetic bead columns (Miltenyi Biotec) were stimulated with anti‐CD3/CD28 beads and recombinant IL (rIL)‐2 for 3 days according to the manufacturer's instructions. The IL‐10 levels in the culture supernatants were detected using a mouse IL‐10 ELISA kit (Life Technologies; Thermo Fisher Scientific). Serum concentrations of total IgE or IL‐4 in OVA‐treated mice were measured using ELISA according to the manufacturer's instructions.

### 2.11 NOD Model

NOD/ShiJcl mice are a spontaneous model of autoimmune diabetes mellitus characterized by hyperglycemia and hyperuricemia due to T cell‐dependent insulitis caused by various factors, including defects in thymic negative selection caused by MHC‐II mutations [18, 19]. Disease onset begins at approximately 18 weeks of age, and 60% of mice develop the disease by 35 weeks of age. Therefore, experimental endpoints were set at 17 weeks of age as the early disease stage and 30 weeks of age as the late disease stage. Mice were provided with a 1.1 M d‐sorbose solution from 7.5 weeks of age, whereas the control group received distilled water. During the treatment period, blood samples were collected every 3 days, and body weight, water consumption, and food intake were measured. Serum was separated from peripheral blood, and blood glucose levels were measured using a microplate assay with a glucose kit (Sysmex, Hyogo, Japan). Persistent hyperglycemia (blood glucose levels ≥ 200 mg/dL recorded for 6 or more consecutive days) was considered indicative of autoimmune diabetes mellitus onset. Peripheral blood was collected into tubes containing EDTA‐2Na at 15 weeks of age, and the peripheral blood, spleen, pancreas, and pancreatic lymph nodes were collected at 17 and 30 weeks of age.

### 2.12 Intracellular Cytokine Staining

For intracellular staining, splenocytes from NOD mice (RRID:IMSR_JCL:JCL:MDB‐0006) were cultured in RPMI‐1640 complete medium at a density of 1 × 10^6^ cells/mL in 96‐well culture plates for 3 h. The cultures were supplemented with phorbol 12‐myristate 13‐acetate (50 ng/mL), ionomycin (250 ng/mL), and 1 μg/mL GolgiPlug simultaneously at the beginning of the culture period. Cells were incubated with anti‐CD16/32 (RRID:AB_312801, clone: 93; BioLegend) to block Fc receptors and prevent nonspecific Ab binding, and with ethidium bromide (Sigma‐Aldrich) for dead cell staining, followed by staining with the following mAbs: APC‐conjugated anti‐CD3 antibody (RRID:AB_2561456, clone: 17A2; BioLegend) and FITC‐conjugated anti‐CD4 antibody (RRID:AB_312691, clone: GK1.5; BioLegend). After staining, cells were fixed, permeabilized with Cytofix/Cytoperm buffer (BD Pharmingen, San Diego, CA), and stained with PE‐conjugated anti‐IFN‐γ antibody (RRID:AB_315402, clone: XMG1.2; BioLegend) or PE‐conjugated Rat IgG2b κ isotype control antibody (RRID:AB_326552, clone: RTK4530; BioLegend). Stained cells were analyzed using the Galios flow cytometry (Beckman Coulter). Data were analyzed using Kaluza analysis software (RRID:SCR_016182, ver. 2.1, Beckman Coulter).

### 2.13 Histopathology

The mouse pancreas was divided into gastric, splenic, and duodenal lobes [20, 21, 22], and pancreatic samples were collected from each lobe. The duodenal lobe, which islets are most abundantly distributed [23], was fixed in 10% neutral buffered formalin, wrapped in 100‐μm mesh, and permeabilized and embedded in paraffin.

Multiple 3‐μm‐thick sections were prepared from the duodenal lobe of the mouse pancreas at 50‐μm intervals. The sections were stained with hematoxylin and eosin (H&E) and scored for lymphocytic infiltration of the pancreatic islets of Langerhans according to a previously reported method [24]. The following criteria were used: 0, no lymphocytes; 1, lymphocytes surrounding the islets but not infiltrating them; 2, lymphocytes occupying less than 25% of the islets; 3, lymphocytes occupying 25%–50% of the islets; 4, lymphocytes occupying more than 50% of the islets; 5, lymphocytes occupying 100% of the islets or complete loss of islets with only remnants detected. Thirty islets from each animal were scored, and the percentage of islets assigned to each grade was determined.

Sections (1 μm thick) were prepared from the lungs of OVA model mice, stained with H&E, and evaluated for the following findings: inflammatory cell infiltration, bronchial epithelial thickening, goblet cell hyperplasia, bronchial smooth muscle thickening, and vascular and peribronchial mucosal edema.

### 2.14 Immunohistochemical Staining

Sections of duodenal lobes (1 mm thick) were immunohistochemically stained for Foxp3. A rabbit anti‐Foxp3 antibody (RRID:AB_2860568, ab215206; Abcam, Cambridge, UK) was used as the primary antibody. After deparaffinization and dehydration, sections were heat treated with Histofine Antigen Activating Solution (pH 9.0) (Nichirei Bioscience, Tokyo, Japan) at 121°C for 20 min. To eliminate endogenous peroxidase activity, sections were immersed in methanol containing 0.3% hydrogen peroxide for 30 min. After blocking nonspecific binding with 1% BSA, sections were incubated overnight at 4°C with the primary antibody. Secondary antibody (RRID:AB_2336536, ImmPRESS Reagent anti‐rabbit IgG, Vector Laboratories, Newark, CA) conjugated with a peroxidase polymer was applied at 24°C for 30 min, followed by incubation with the substrate solution 3,3′‐diaminobenzidine tetrahydrochloride (DAB; Dako, Santa Clara, CA). Meyer's hematoxylin was used for counterstaining.

### 2.15 Cell Viability Measurements

Tregs (CD4^+^CD25^+^) and non‐Tregs (CD4^+^CD25^−^) were isolated from the spleens of male C57BL/6J (11 weeks old) using the MACS Pan T Cell Isolation Kit II and the CD4^+^CD25^+^ Regulatory T Cell Isolation Kit (Miltenyi Biotec).

Cells were seeded at 1 × 10^5^ cells/well in flat‐bottom 96‐well plates and stimulated with recombinant human IL‐2 (30 IU/mL) and anti‐CD3/CD28 in the presence of 25 mM monosaccharides. Cells were cultured at 37°C in 5% CO_2_. After 24 and 72 h of stimulation, cells were harvested, stained with trypan blue (Fujifilm Wako Pure Chemicals) to assess cell viability, and viable cells were quantified.

### 2.16 Intracellular Glucose Metabolite Measurements

T cells were isolated from the spleens of male C57BL/6J (11 weeks old) using the MACS Pan T Cell Isolation Kit II (Miltenyi Biotec).

Metabolites were extracted from the cells according to previously reported methods [24, 25, 26]. T cells were seeded at 2 × 10^6^ cells/well in flat‐bottom 96‐well plates and cultured. After a 24‐h pre‐incubation period to stabilize metabolism, d‐sorbose was added to a final concentration of 25 mM, and the cells were cultured for an additional 6 h. For the control group, complete RPMI medium was used instead of d‐sorbose. Cells were collected and washed three times with PBS (800 × *g*, 4°C, 5 min) to completely remove the medium. Distilled water was added to the cell pellet, mixed thoroughly, and the cells were disrupted using an ultrasonic homogenizer (VP‐050 N; Titec, Aichi, Japan). Equal volumes of methanol and distilled water were then added, and the supernatant was collected after centrifugation (20 000 × *g*, 4°C, 10 min). The supernatant was stored at −80°C until analysis. To 200 μL of the thawed supernatant, 30 μL of 10 μM 2‐morpholinoethanesulfonic acid was added. The samples were dried under reduced pressure, redissolved in 30 μL of water, and then subjected to liquid chromatography–tandem mass spectrometry (LC–MS/MS).

### 2.17 LC–MS/MS

To comprehensively investigate glucose metabolism, seven metabolites representing divergent metabolic pathways were measured. An LCMS‐8060 mass spectrometer (Shimadzu Corporation, Kyoto, Japan) coupled to a Nexcera HPLC system, was used for the analysis. HPLC was performed using an Inert Sustain PFP column (150 × 2.1 mm, 1.9 μm; GL Science, Tokyo, Japan) maintained at 40°C, with 0.1% formic acid solution as the initial mobile phase. Cell extracts (1 μL) were injected, and metabolites were separated using a mobile phase gradient at a flow rate of 300 μL/min with the concentration of 0.1% formic acid in acetonitrile (Fujifilm Wako) increasing to 25% over 3 min. The total analysis time was 6 min. Data were acquired using LabSolutions software ver. 2.2 (RRID:SCR_018241), and metabolite peak areas were determined. Conversion efficiencies were calculated from the quantitative metabolite values and used to determine metabolic enzyme activities.

### 2.18 Effect of Glucose Metabolism Inhibition on Treg Differentiation

To investigate the effect of intracellular metabolism on Treg differentiation, Treg differentiation cultures were performed in the presence of glucose metabolism inhibitors. CD3^+^ T cells were isolated from the spleens of male C57BL/6N mice (9 weeks old, RRID:IMSR_RJ:C57BL‐6NRJ). Isolated CD3^+^ T cells were seeded at 1 × 10^5^ cells/well in flat‐bottom 96‐well plates, stimulated with recombinant human IL‐2 and anti‐CD3/CD28 in the presence of various inhibitors, and cultured at 37°C with 5% CO_2_. The final concentrations of the inhibitors were set at approximately 10 times the half‐maximal inhibitory concentration (IC₅₀) of each inhibitor: KAN was used at 2 μM, 6‐AT at 5 μM, and DON at 2 μM. For the vehicle group, a complete RPMI medium containing the same concentration of DMSO as the inhibitor solvent was used.

To assess the contribution of glutamine availability to Treg differentiation, cells were cultured in either standard RPMI‐1640 medium containing 2 mM L‐glutamine or glutamine‐free RPMI‐1640 medium supplemented with 10% dialyzed FBS (HyClone; Cytiva, Tokyo, Japan). DON was added at the indicated concentrations at the start of culture. To determine whether Treg induction depended on inhibition of the hexosamine biosynthetic pathway (HBP), naïve CD4^+^ T cells isolated from the spleens of male C57BL/6N mice were cultured under induced Treg (iTreg)‐polarizing conditions in the presence or absence of DON. N‐acetyl‐d‐glucosamine (GlcNAc) was added to the culture medium at the indicated concentrations (5 or 10 mM) at the start of culture. After 72 h, cells were harvested and stained for CD4 and Foxp3 expression, and the frequency of Treg cells was analyzed by flow cytometry.

### 2.19 Statistical Analysis

Statistical analyses were performed using GraphPad Prism software (version 6; RRID:SCR_002798; GraphPad Software, San Diego, CA, USA). All data are presented as the mean ± standard deviation. Comparisons between two groups were performed using the unpaired Student's *t*‐test. Comparisons among three or more groups were performed using one‐way or two‐way analysis of variance, followed by Šídák's multiple‐comparison test or Dunnett's multiple‐comparison test, as appropriate. A *p* value < 0.05 was considered statistically significant.

## 3 Results

### 3.1 d‐Sorbose Promotes Ex Vivo Treg Differentiation

**FIGURE 1:**
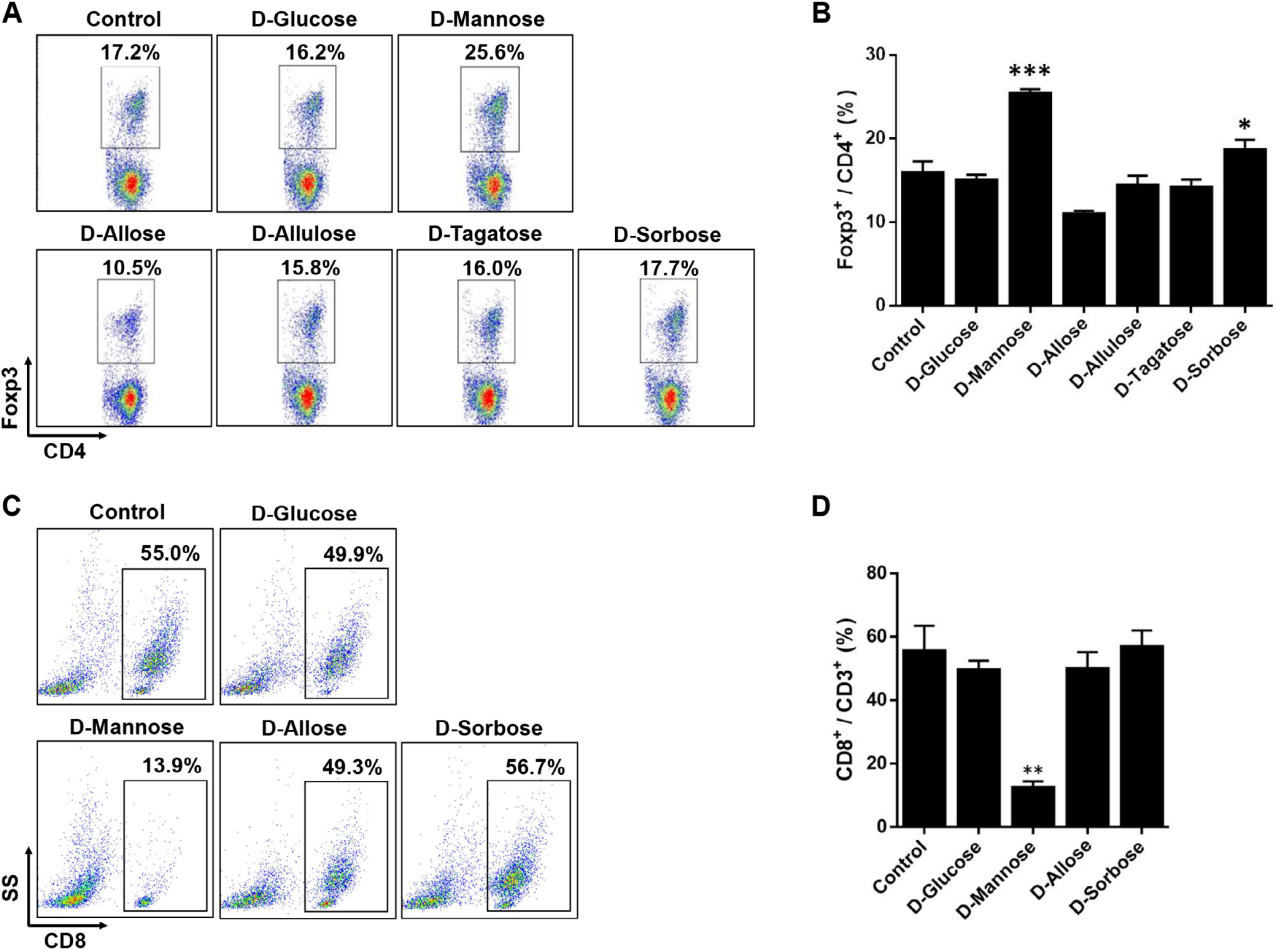
Rare sugars induce Treg ex vivo. (A, B) Splenic naïve CD4^+^ T cells were stimulated with anti‐CD3/CD28 beads, recombinant transforming growth factor‐beta 1 (rTGF‐β1), and recombinant interleukin‐2 (rIL‐2) in the presence or absence of the indicated monosaccharides (25 mM). Five days after stimulation, the frequency of CD3^+^CD4^+^Foxp3^+^Tregs was analyzed by flow cytometry (FACS). Representative dot plots (A) and cumulative data graphical summary (B) are shown (*n* = 3). (C, D) Splenic CD3^+^ T cells were stimulated with anti‐CD3/CD28 beads and rIL‐2 in the presence or absence of the indicated monosaccharides (25 mM). Six days after stimulation, the frequency of CD8^+^ T cells was analyzed by flow cytometry (FACS). Representative dot plots (C) and a graphical summary of the cumulative data (D) are shown (*n* = 3). Data are presented as the mean ± standard deviation. Statistical analyses were performed using one‐way analysis of variance followed by Dunnett's multiple‐comparison test. *p < 0.05, **p < 0.01, ***p < 0.001 vs. d‐glucose.

To assess the potential of rare sugars to induce Treg differentiation ex vivo, splenic naïve CD4^+^ T cells were cultured with various rare sugars and analyzed using flow cytometry. Compared with the d‐glucose group, d‐sorbose significantly increased Treg frequency to a level comparable to that induced by d‐mannose, a known Treg‐inducing sugar (Figure 1A,B). In contrast, only d‐mannose significantly reduced the frequency of CD8^+^ T cells (*p* < 0.01), whereas d‐sorbose had no such effect (Figure 1C,D). These findings demonstrate that d‐sorbose directly promotes Treg differentiation.

### 3.2 d‐Sorbose Administration Ameliorates OVA‐Induced Airway Inflammation via Treg Induction

Continuous administration of 1.1 M d‐sorbose initially caused body weight loss accompanied by reduced water and food intake. These parameters began to recover by Day 5, and body weight and food intake returned to control levels by Day 10, although water intake remained reduced until Day 28 (Figure S2a). On Day 28, serum levels of TP, ALT, and glucose were unchanged between the groups, whereas Cre levels were modestly elevated in the d‐sorbose group but remained within the physiological range (Figure S2b).

**FIGURE 2:**
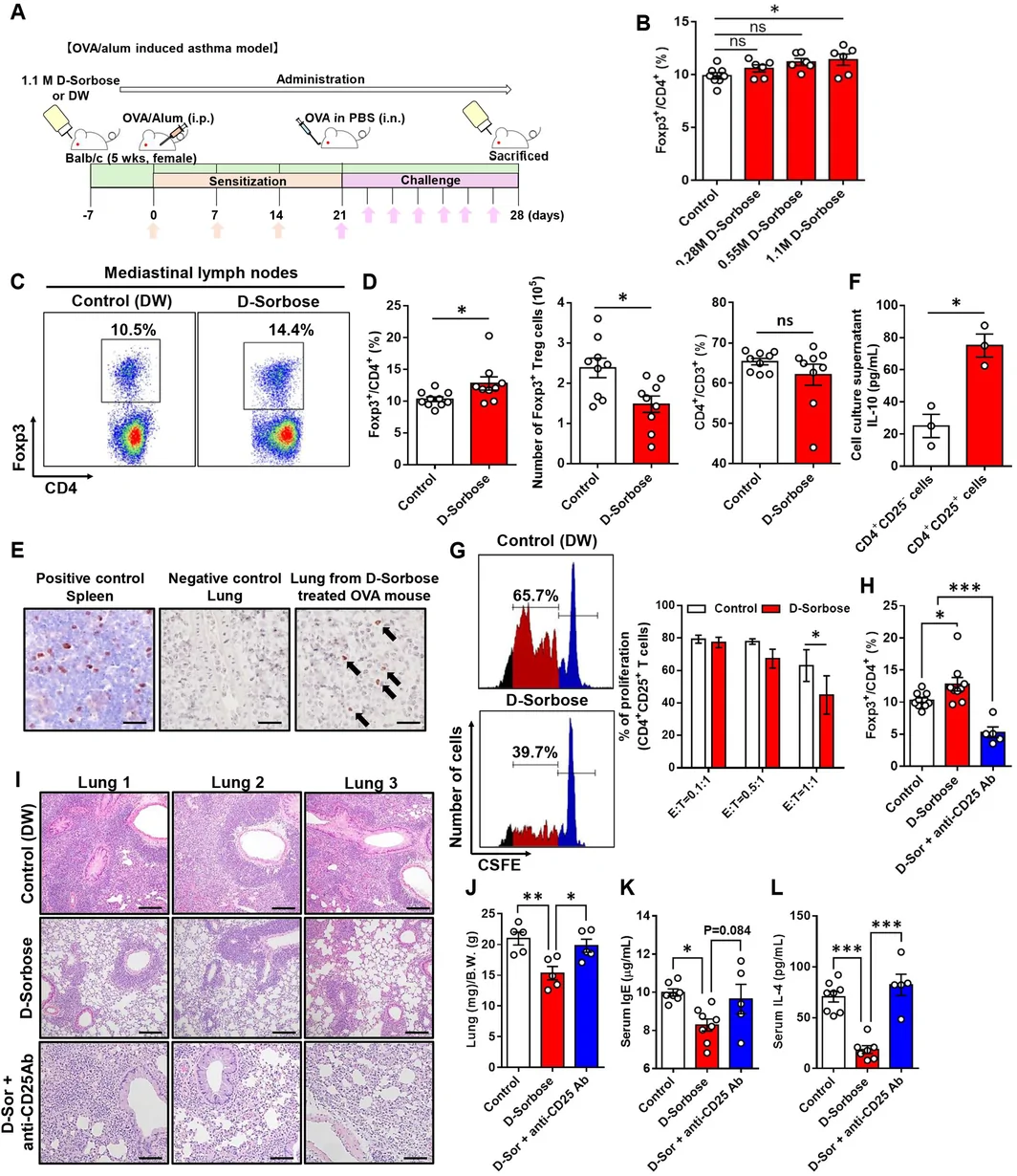
d‐sorbose suppresses OVA‐induced asthma development. (A) BALB/c mice were sensitized by intraperitoneal (i.p.) injection of ovalbumin (OVA) or phosphate buffered saline (PBS) with aluminum hydroxide on Days 0, 7, and 14. From Days 21 to 27, the mice were challenged once daily by intranasal (i.n.) administration of OVA or PBS. Samples were collected on Day 28. (B) Dose‐dependent changes in Treg frequency in the mediastinal lymph nodes (MLNs) of OVA mice treated with d‐sorbose. (C, D) The frequency and absolute number of Tregs and CD4^+^ T cells in the MLNs of OVA mice receiving the indicated treatments were analyzed by flow cytometry (FACS). Representative dot plots (C) and a graphical summary of the cumulative data (D) are shown. (E) Representative Foxp3 immunostaining of lung tissue from OVA mice. Scale bar = 100 μm. (F) CD4^+^CD25^+^ T cells or CD4^+^CD25^−^ T cells isolated from the MLNs of OVA mice were stimulated with anti‐CD3/CD28 beads and recombinant interleukin‐2 (rIL‐2) for 3 days. Interleukin‐10 (IL‐10) levels in the culture supernatants were measured by ELISA (*n* = 3 independent experiments). (G) Carboxyfluorescein succinimidyl ester (CFSE)‐labeled CD4^+^CD25^−^ T cells isolated from the MLNs of OVA mice were stimulated with anti‐CD3/CD28 Dynabeads and co‐cultured with CD4^+^CD25^+^ Tregs isolated from the MLNs of OVA mice receiving the indicated treatments at effector‐to‐target ratios of 1:1, 0.5:1, and 0.1:1. After 3 days, CFSE‐labeled CD4^+^CD25^−^ T cells were harvested, stained with anti‐CD3 and 7‐aminoactinomycin D (7‐AAD), and analyzed by flow cytometry (*n* = 3). Representative histograms and a graphical summary of the cumulative data are shown. (H) Treg frequency in the MLNs of OVA mice receiving the indicated treatments was analyzed by flow cytometry (FACS). A graphical summary of the cumulative data is shown. (I, J) Representative hematoxylin and eosin (H&E)‐stained lung sections and the lung‐to‐body weight ratio in OVA mice receiving the indicated treatments. Scale bar = 100 μm. (K, L) Serum immunoglobulin E (IgE) and IL‐4 levels were measured by ELISA. Data are presented as the mean ± standard deviation. Statistical analyses were performed using one‐way analysis of variance (ANOVA) followed by Dunnett's multiple‐comparison test (B, H, J, K, L) or the unpaired Student's *t*‐test (D, F). *p < 0.05, **p < 0.01, ***p < 0.001 vs. the indicated groups.

A murine model of OVA‐induced airway inflammation was used to investigate the in vivo effects of d‐sorbose (Figure 2A). In untreated mice, d‐sorbose did not affect Treg frequency (Figure S3c,d). However, in OVA‐challenged mice, d‐sorbose increased Treg frequency in a dose‐dependent manner, with a significant effect observed at 1.1 M (Figure 2B, *p* < 0.05). In addition, Treg frequency was significantly increased in both mediastinal lymph nodes and inflamed lung tissue (Figure 2C,E). Intrigued by the finding that the absolute number of Tregs was lower in the d‐sorbose group, likely reflecting an overall reduction in total cellular infiltration, we assessed whether d‐sorbose affected other T‐cell compartments. Notably, d‐sorbose administration did not alter conventional CD4^+^ T‐cell subsets in the mediastinal lymph nodes (Figure 2D). To determine whether these d‐sorbose‐induced Tregs retained intact suppressive capacity, we performed in vitro suppression assays by co‐culturing Tregs and effector T cells at varying ratios. Tregs isolated from both the control and d‐sorbose‐treated groups exhibited potent suppressive activity; however, Tregs from the d‐sorbose‐treated group demonstrated even greater suppressive activity (Figure 2G, *p* < 0.05). Consistent with their functional maturity, d‐sorbose‐induced Tregs produced significantly higher levels of IL‐10 relative to non‐Treg CD4^+^ T cells (Figure 2F, *p* < 0.05). Concomitantly, d‐sorbose administration markedly ameliorated OVA‐induced airway inflammation (Figure 2H–L). Crucially, systemic depletion of Tregs using an anti‐CD25 neutralizing antibody completely abrogated the protective effects of d‐sorbose. Collectively, these findings demonstrate that oral d‐sorbose supplementation selectively promotes the induction of functional Tregs, thereby attenuating allergic airway inflammation in vivo.

### 3.3 d‐Sorbose Prevents Autoimmune Diabetes by Promoting Treg Induction in Pancreatic Islets

**FIGURE 3:**
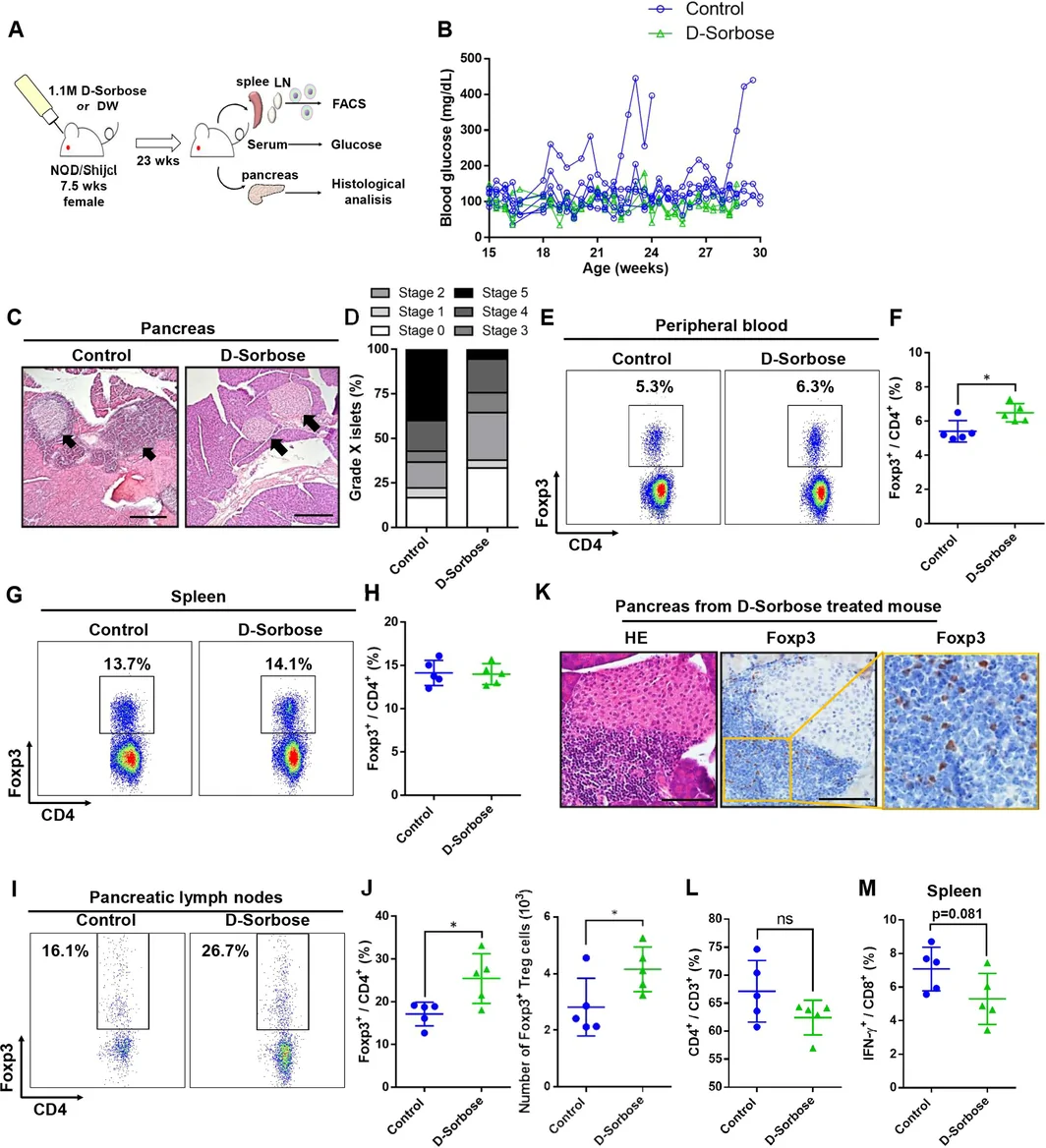
d‐sorbose suppresses autoimmune diabetes development. (A) NOD/ShiJcl mice (7.5 weeks old) were given 1.1 M d‐sorbose solution (*n* = 5) or distilled water (control, *n* = 5) ad libitum, and various analyses were performed using blood and tissue samples collected from the indicated organs. (B) Time course of changes in serum glucose levels. (C, D) Representative hematoxylin and eosin (H&E)‐stained pancreatic sections and inflammatory stages of the islets of Langerhans at 30 weeks of age. Arrows indicate the islets of Langerhans. Scale bar = 100 μm. (E–J) The frequency and absolute number of Tregs in peripheral blood, pancreatic lymph nodes, and spleen of 17‐week‐old NOD/ShiJcl mice were analyzed by flow cytometry (FACS). Representative dot plots and graphical summaries of the cumulative data are shown. (K) Representative H&E staining and Foxp3 immunostaining of pancreatic islets from 17‐week‐old NOD/ShiJcl mice. Scale bar = 100 μm. (L, M) The frequencies of CD3^+^CD4^+^ T cells and interferon‐gamma (IFN‐γ)^+^CD8^+^ T cells in the pancreatic lymph nodes of 17‐week‐old NOD/ShiJcl mice were analyzed by flow cytometry (FACS). Graphical summaries of the cumulative data are shown. Data are presented as the mean ± standard deviation (*n* = 3–5). Statistical analyses were performed using the unpaired Student's *t*‐test. *p < 0.05 vs. control.

Encouraged by the protective effect of d‐sorbose on airway inflammation, we examined whether d‐sorbose‐induced Treg generation also affects autoimmune pathology. In NOD/ShiJcl mice, a spontaneous model of Type 1 diabetes, disease incidence and Treg induction were evaluated following d‐sorbose administration (Figure 3A). Interestingly, 60% of the untreated control mice developed hyperglycemia, whereas the d‐sorbose‐treated mice remained normoglycemic throughout the experimental period (Figure 3B). Histological analysis revealed reduced lymphocyte infiltration and maintained islet integrity in the d‐sorbose group compared with the control group (Figure 3C,D). The frequency and absolute number of Tregs were significantly increased in the peripheral blood (*p* < 0.05) and pancreatic lymph nodes (*p* < 0.05) during the early stage of disease, whereas no significant differences were observed in the spleen (Figure 3E–J). Importantly, Treg infiltration into the pancreatic islets was detected in the d‐sorbose group during the early stage of disease progression (Figure 3K). At this stage, d‐sorbose administration did not affect conventional CD4^+^ T‐cell subsets in the pancreatic lymph nodes (Figure 3L), and IFN‐γ production by splenic CD8^+^ T cells showed a decreasing trend compared with the control group (Figure 3M). These findings suggest that d‐sorbose promotes Treg induction in the pancreatic lymph nodes and their infiltration into the pancreatic islets, thereby protecting against autoimmune diabetes.

### 3.4 d‐Sorbose Does not Alter Treg Survival

To exclude the possibility that the increased Treg frequency resulted from reduced survival of non‐Treg CD4^+^ T cells, we compared cell viability between the d‐sorbose and d‐glucose‐treated groups. No significant differences in cell viability were observed among T‐cell subsets or across time points (Figure S3). Thus, the increased Treg frequency induced by d‐sorbose reflected de novo differentiation rather than altered cell survival.

### 3.5 d‐Sorbose Promotes Treg Differentiation Through Metabolic Reprogramming Involving Glucose and Glutamine Metabolism

**FIGURE 4:**
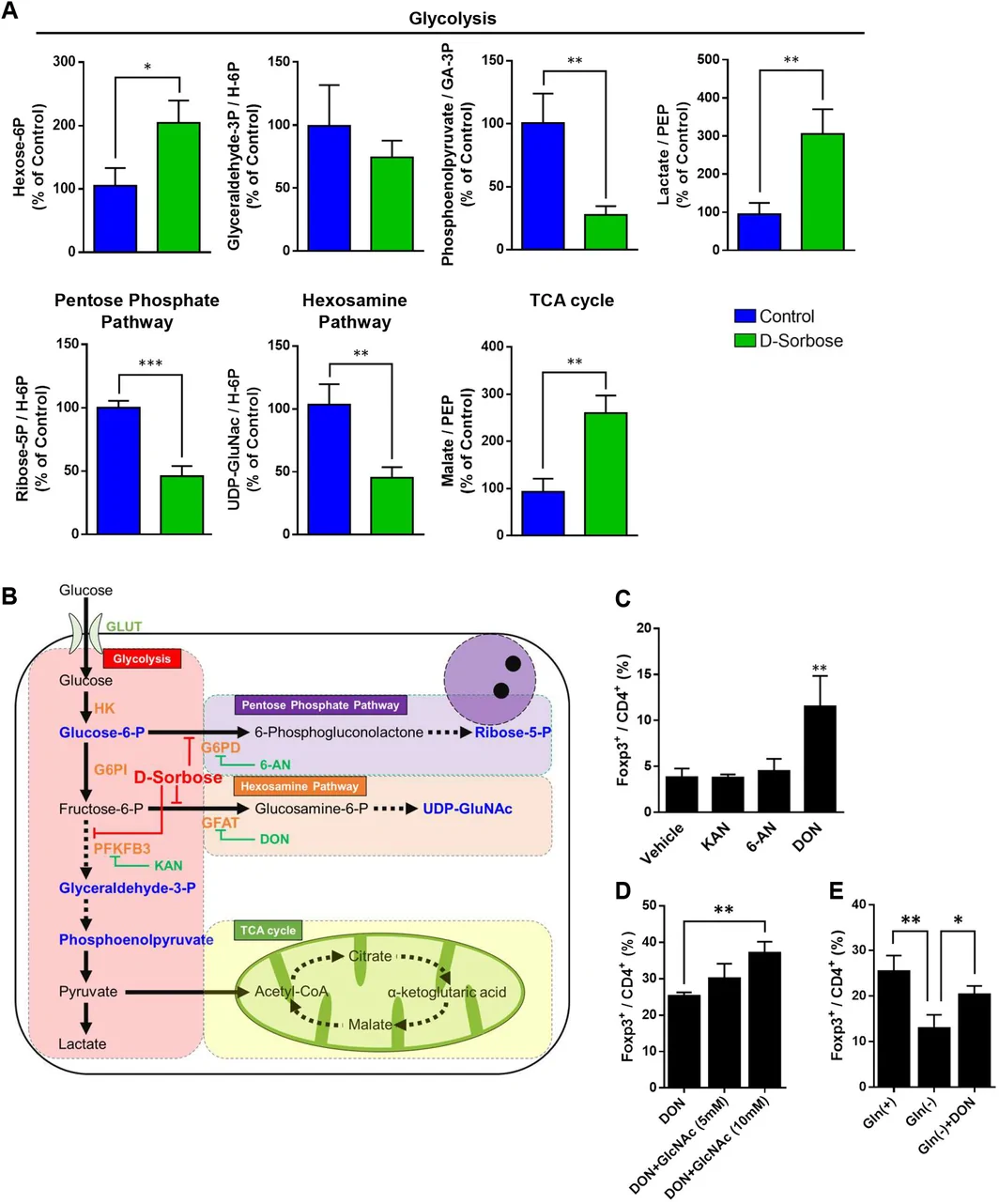
d‐sorbose may induce Treg differentiation via hexosamine pathway inhibition. (A) Splenic T cells were seeded at 2 × 10^6^ cells/well. After 24 h of culture, 25 mM d‐sorbose or complete Roswell Park Memorial Institute (RPMI)‐1640 medium (control) was added. After an additional 6 h, intracellular glucose metabolites were quantified by liquid chromatography–tandem mass spectrometry (LC–MS/MS). The ratios of each metabolite were used to estimate metabolic enzyme activities. (B) Schematic diagram of glycolysis, the tricarboxylic acid (TCA) cycle, the pentose phosphate pathway (PPP), and the hexosamine biosynthetic pathway (HBP). Metabolites that were significantly decreased in the d‐sorbose group are shown in blue. (C) Splenic T cells isolated from C57BL/6N mice were stimulated with anti‐CD3/CD28 beads and recombinant interleukin‐2 (rIL‐2) in the presence or absence of glucose metabolism inhibitors (KAN, 2 μM; 6‐AN, 5 μM; DON, 2 μM). After 6 days of culture, Treg frequency was analyzed by flow cytometry (FACS). A graphical summary of the cumulative data is shown. 6‐AN, 6‐aminonicotinamide; DON, 6‐diazo‐5‐oxo‐L‐norleucine; G6PD, glucose‐6‐phosphate dehydrogenase; G6PI, glucose‐6‐phosphate isomerase; GFAT, glutamine‐fructose‐6‐phosphate aminotransferase; HK, hexokinase; KAN, KAN0438757; PFKFB3, 6‐phosphofructo‐2‐kinase/fructose‐2, 6‐bisphosphatase 3. (D, E) Splenic naïve CD4^+^ T cells isolated from C57BL/6N mice were stimulated with anti‐CD3/CD28 beads, recombinant transforming growth factor‐beta 1 (rTGF‐β1; 2 ng/mL), and rIL‐2 (20 IU/mL) in the presence or absence of DON, N‐acetyl‐d‐glucosamine (GlcNAc), and glutamine (Gln). After 3 days of culture, Treg frequency was analyzed by flow cytometry (FACS). Graphical summaries of the cumulative data are shown. Data are presented as the mean ± standard deviation (*n* = 3). Statistical analyses were performed using the unpaired Student's *t*‐test (A) and one‐way analysis of variance (ANOVA) followed by Dunnett's multiple‐comparison test (C–E). *p < 0.05, **p < 0.01, ***p < 0.001 vs. the indicated group or vehicle.

Intracellular metabolites were quantified to elucidate the metabolic mechanisms underlying d‐sorbose‐mediated Treg induction. d‐Sorbose significantly increased hexose‐6‐phosphate levels (*p* < 0.05) while reducing phosphoenolpyruvate (glycolysis, *p* < 0.01), ribose‐5‐phosphate (pentose phosphate pathway [PPP], *p* < 0.001), and UDP‐GlcNAc (hexosamine biosynthetic pathway [HBP], *p* < 0.01) levels (Figure 4A). Conversely, d‐sorbose significantly increased malate levels, a tricarboxylic acid (TCA) cycle intermediate (*p* < 0.01). These data suggest that d‐sorbose inhibits key enzymatic activities in glycolysis, the PPP, and the HBP (Figure 4B).

Next, we examined Treg induction following selective inhibition of metabolic pathways branching from glucose‐6‐phosphate. Inhibition of 6‐phosphofructo‐2‐kinase/fructose‐2, 6‐bisphosphatase 3 (PFKFB3), a key regulator of glycolysis, or glucose‐6‐phosphate dehydrogenase (G6PD), the rate‐limiting enzyme of the PPP, did not affect Treg frequency. In contrast, inhibition of glutamine–fructose‐6‐phosphate aminotransferase (GFAT), the rate‐limiting enzyme of the HBP, using DON significantly increased Treg frequency (Figure 4C, *p* < 0.01). To investigate the underlying mechanism, we assessed Treg differentiation under GFAT inhibition by DON in the presence of N‐acetyl‐d‐glucosamine (GlcNAc) supplementation or glutamine restriction. Compared with DON treatment alone, Treg frequency increased significantly in a GlcNAc concentration‐dependent manner. Furthermore, compared with cells differentiated in medium containing 2 mM glutamine, Treg frequency was significantly reduced in glutamine‐free medium. Notably, the reduction in Treg frequency observed under glutamine‐free conditions was restored by DON treatment. Collectively, these findings suggest that d‐sorbose promotes Treg differentiation through metabolic reprogramming involving altered glucose partitioning and glutamine metabolism, rather than through suppression of the HBP alone.

## 4 Discussion

This study demonstrates that d‐sorbose administration promotes Treg differentiation by modulating intracellular carbohydrate metabolism, thereby suppressing multiple pathological conditions.

OVA‐induced airway inflammation and autoimmune diabetes are prototypical allergic and autoimmune diseases, respectively. During disease progression, effector T cells migrate from the circulation to inflamed tissues, where activated Tregs suppress their proliferation and cytokine production [27]. Although the inhibitory effect of rare sugars on autoimmune diabetes has previously been reported using l‐sorbose, an epimer of d‐sorbose, the underlying mechanism remains unclear.

In the present study, continuous d‐sorbose administration significantly increased the proportion of IL‐10‐producing Tregs within the regional lymph nodes of mice with OVA‐induced airway inflammation or autoimmune diabetes, leading to amelioration of disease pathology. In the OVA‐induced airway inflammation model, the increased Treg frequency should be interpreted in the context of a marked reduction in total mediastinal lymph node cellularity. Although the absolute number of Tregs decreased, the reduction in non‐Treg immune cells appeared to be greater, resulting in an increased proportion of Tregs. This finding suggests that d‐sorbose suppresses the overall inflammatory expansion of lymph node cells while relatively preserving the Treg compartment. In contrast, both the frequency and absolute number of Tregs increased in NOD mice, indicating that the effects of d‐sorbose on Treg homeostasis may differ depending on the immunological context and chronicity of inflammation. Since d‐sorbose did not affect the survival of any T‐cell subset, the increased Treg frequency is likely attributable to enhanced differentiation rather than selective expansion. Tregs arise either as induced Tregs (iTregs), which differentiate from naïve T cells in the periphery, or as naturally occurring Tregs (nTregs), which develop in the thymus. Upon anti‐CD3/CD28 and IL‐2 stimulation, d‐sorbose induced the differentiation of splenic T cells into Tregs, suggesting that d‐sorbose acts directly on T cells to promote iTreg differentiation. Conversely, under steady‐state conditions, d‐sorbose did not affect Treg frequency, indicating that cytokine signaling is required for this process. IL‐2 is particularly essential for Treg differentiation and maintenance, with iTregs exhibiting greater sensitivity to IL‐2 than nTregs [28, 29]. Thus, our findings support a model in which d‐sorbose promotes IL‐2–dependent iTreg differentiation.

The preferential effect of d‐sorbose on Treg differentiation may be explained by the distinct metabolic requirements of regulatory and effector T cells. Effector T cells, particularly Th1 and Th17 cells, rely heavily on aerobic glycolysis to support proliferation and inflammatory cytokine production, whereas Tregs preferentially utilize oxidative phosphorylation and fatty acid oxidation and are less dependent on glycolytic metabolism [30, 31]. Previous studies have demonstrated that inhibition of glycolysis or mammalian target of rapamycin signaling selectively promotes Treg differentiation while suppressing effector T‐cell responses [32]. Therefore, the metabolic perturbation induced by d‐sorbose may create an intracellular environment that is relatively disadvantageous for glycolysis‐dependent effector T cells but permissive for Treg differentiation and maintenance. Although the precise transporter‐mediated mechanisms remain unclear, our findings are consistent with the established concept that metabolic restriction preferentially favors regulatory T‐cell differentiation over glycolysis‐dependent effector T‐cell responses.

Mechanistically, this study demonstrates that d‐sorbose orchestrates a multi‐pathway metabolic reprogramming in naïve CD4^+^ T cells, ultimately promoting de novo Treg differentiation. Under standard T‐cell receptor activation, conventional T cells rely heavily on aerobic glycolysis and glutaminolysis [26, 33]. These high‐flux pathways cooperatively fuel the HBP to synthesize UDP‐GlcNAc, which serves as a critical substrate for protein O‐GlcNAcylation and N‐glycan branching. Although genetic ablation of O‐linked N‐acetylglucosamine transferase (OGT) or disruption of HBP signaling has been reported to compromise Foxp3 stability and suppressive function [34, 35], our finding that d‐sorbose‐mediated metabolic constraint enhances Treg differentiation are not necessarily contradictory. Our metabolomic profile revealed that d‐sorbose suppressed glycolytic intermediates (e.g., phosphoenolpyruvate) and downstream HBP metabolites (e.g., UDP‐GlcNAc), while increasing the tricarboxylic acid (TCA) cycle intermediate malate. The accumulation of malate further supports a metabolic shift toward oxidative phosphorylation, which is highly compatible with the energetic requirements of differentiating Tregs. In activated T cells, excessive glycolysis can paradoxically limit Treg generation by driving differentiation toward a pro‐inflammatory Th17 or Th1 phenotype, as Foxp3 expression requires a metabolic shift from high glycolytic flux toward oxidative phosphorylation (OXPHOS) and fatty acid oxidation [26, 36]. The regulatory role of glutamine and HBP partitioning is further clarified by our in vitro assays using the GFAT inhibitor DON. Although GFAT inhibition restricts the de novo HBP pathway, previous studies have shown that limiting glutamine availability or restricting its conversion to α‐ketoglutarate (αKG) through glutaminolysis strongly promotes the differentiation of naïve CD4^+^ T cells into Tregs rather than Th1 cells [37]. Indeed, an altered intracellular αKG‐to‐succinate ratio, or excessive αKG generated during active glutaminolysis, acts as a metabolic checkpoint that epigenetically suppresses Foxp3 induction [38]. The observation that d‐sorbose reduces glycolytic activity, together with our findings that glutamine deprivation or DON‐mediated GFAT inhibition promotes Treg differentiation, strongly suggests that d‐sorbose acts by limiting excessive glycolytic and glutaminolytic flux, thereby optimizing the intracellular metabolic state for Treg commitment. Crucially, our rescue experiments showed that supplementation with N‐acetyl‐d‐glucosamine (GlcNAc) in DON‐treated cells, or restoration of glutamine under DON treatment in glutamine‐deprived cells, significantly altered Treg frequencies, demonstrating that Treg differentiation is not merely a passive response to HBP suppression. Instead, d‐sorbose promotes Treg differentiation through coordinated metabolic reprogramming that optimizes glucose partitioning and glutamine catabolism, thereby avoiding the pro‐inflammatory metabolic program [33] and establishing an intracellular environment permissive for functional Foxp3 expression.

Previous studies have evaluated the effects of d‐sorbose and d‐sorbose‐containing syrups in humans and rodents [39, 40]; however, long‐term safety data remain limited. In the present study, we evaluated the physiological effects of continuous d‐sorbose administration in mice. After 1 month of continuous treatment, no pathological changes were observed in body weight, renal or hepatic function, blood glucose levels, or nutritional parameters. The transient body weight loss observed during the early phase of treatment was associated with reduced food and water intake. Although the underlying mechanism remains unclear, body weight loss was not observed at a d‐sorbose concentration of 0.28 M, which did not affect food or water intake (Figure S4). Therefore, a gradual dose‐escalation approach, beginning at 0.28 M and increasing to 1.1 M, may improve tolerability. Furthermore, in ex vivo experiments, d‐mannose induced both Treg expansion and a reduction in CD8^+^ T cells, whereas d‐sorbose increased Treg frequency without affecting CD8^+^ T cells. Thus, d‐sorbose may retain the Treg‐inducing capacity of d‐mannose while avoiding its potential effects on CD8^+^ T cells.

This study has several limitations. First, we focused primarily on CD4^+^ T‐cell subsets and their glucose metabolism as the main targets of d‐sorbose. However, the potential effects of d‐sorbose on other immune cell types and metabolic pathways have not yet been investigated. In addition, the specific metabolic enzymes inhibited by d‐sorbose, as well as its direct inhibitory effects on enzyme activity and stereochemical interactions, were not identified. Elucidating these aspects is essential for clarifying the mechanisms by which d‐sorbose promotes Treg differentiation and immune regulation.

## 5 Conclusion

We identified d‐sorbose as a novel immunoregulatory sugar that promotes Treg differentiation by modulating intracellular glucose metabolism, particularly through coordinated regulation of glucose partitioning and glutamine metabolism. Thus, d‐sorbose has potential as a dietary or therapeutic agent for the treatment of inflammatory and autoimmune diseases. Future studies addressing these limitations may further facilitate the clinical translation of these findings.

## Author Contributions

M.H., Y.K., H.I., and K.S. conducted this study. F.S., M.H., H.Y., S.Y., R.T., N.S., N.M., C.T., K.Y., and H.T. performed the experiments. F.S. and M.H. were responsible for data integrity and analysis. F.S., M.H., H.Y., S.Y., R.T., N.S., N.M., C.T., K.Y., Y.K., H.T., H.I., and K.S. discussed the results. F.S., M.H., and H.T. wrote the manuscript. M.H. was primarily responsible for the final content. All the authors reviewed the manuscript.

## Funding

This study was supported by JSPS KAKENHI (Grant Number 25K14944), Fujita Health University Grant, and Matsutani Chemical Industry Co., Ltd.

## Conflicts of Interest

Kyoka Yamazaki and Yuka Kishimoto are employees of the Matsutani Chemical Industry Co. Ltd. The remaining authors declare no competing financial interests.

## Supporting information


**Table S1:** Characteristics of the monosaccharides used in the experiment.

**Figure S1:** Gating strategy for flow cytometry assay.

**Figure S2:** Safety evaluation of mice treated with 1.1 M d‐sorbose.

**Figure S3:** d‐Sorbose does not affect Treg cell viability.

**Figure S4:** Safety evaluation of mice treated with 0.28 M d‐sorbose.

## Data Availability

The data supporting the findings of this study are available from the corresponding author upon reasonable request.
