# Supplementary material for: d‐Sorbose Promotes Regulatory T‐Cell Differentiation and Ameliorates Autoimmune and Allergic Diseases via Hexosamine Biosynthetic Pathway Inhibition
Source: FASEB J. 2026 Aug 28;40(17):e72252. doi: 10.1096/fj.202601110R (PMC13524200; doi:10.1096/fj.202601110R)
Supplement: Supplementary file 1 [file FSB2-40-e72252-s002.pdf]

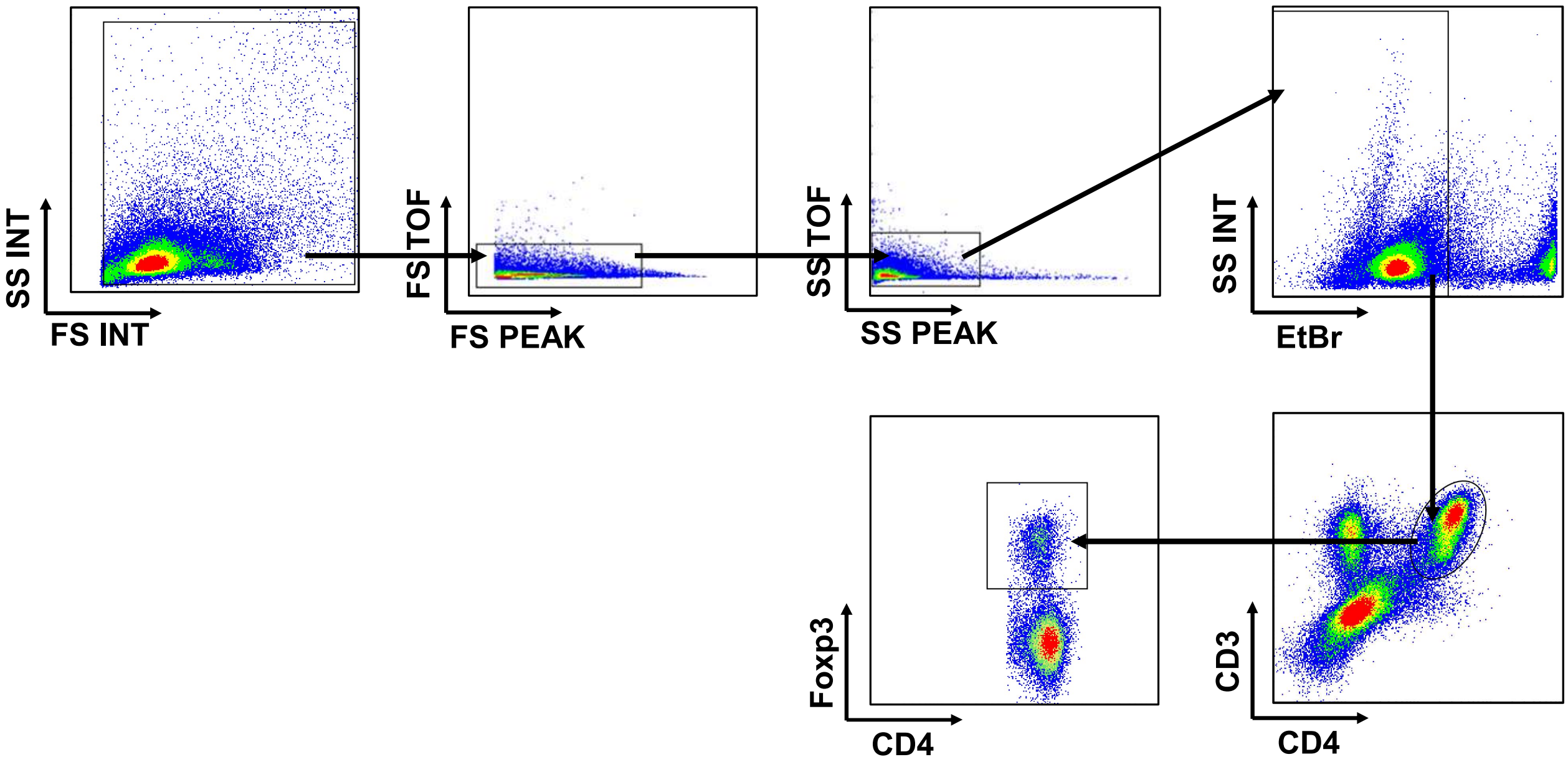

Supplemental figure 1. Gating strategy for flow cytometry assay

Gating strategy used to identify Tregs by flow cytometry. From left to right and top to bottom: (a) cell-like events were identified based on forward scatter area (FSC-A) versus side scatter area (SSC-A); (b, c) singlets were identified using forward scatter area (FSC-A) versus forward scatter height (FSC-H); (d) live cells were identified by excluding ethidium bromide (EtBr)-positive cells; and (e) T cells were identified as $CD3^{+}CD4^{+}$ cells, and Tregs were defined as $CD4^{+}Foxp3^{+}$ cells.
