# Supplementary material for: d‐Sorbose Promotes Regulatory T‐Cell Differentiation and Ameliorates Autoimmune and Allergic Diseases via Hexosamine Biosynthetic Pathway Inhibition
Source: FASEB J. 2026 Aug 28;40(17):e72252. doi: 10.1096/fj.202601110R (PMC13524200; doi:10.1096/fj.202601110R)
Supplement: Supplementary file 2 [file FSB2-40-e72252-s003.pdf]

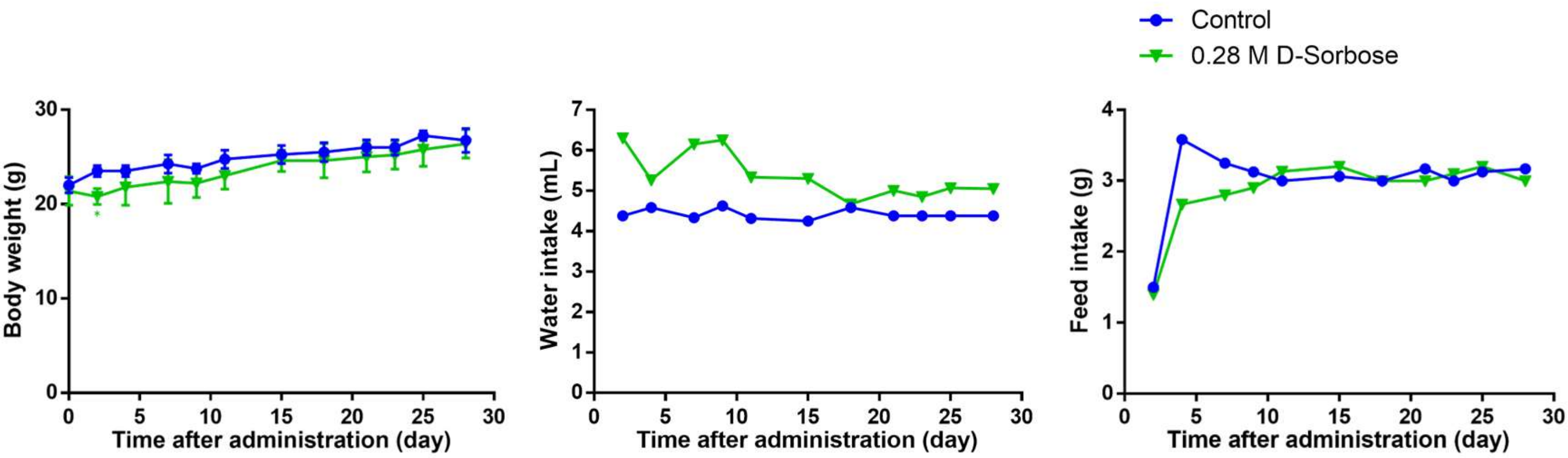

Supplemental figure 4. Safety evaluation of mice treated with 0.28 M d-Sorbose

C57BL/6J mice were given 0.28 M d-sorbose solution or distilled water (control) ad libitum for 4 weeks. Body weight and water and food intake were monitored throughout the treatment period. Water and food intake are presented as the average per cage. Data are presented as the mean ± standard deviation (n = 3–6). Statistical analysis was performed using two-way analysis of variance (ANOVA) followed by Sidak's multiple-comparison test. $p < 0.05$ vs. control.
