# Supplementary material for: d‐Sorbose Promotes Regulatory T‐Cell Differentiation and Ameliorates Autoimmune and Allergic Diseases via Hexosamine Biosynthetic Pathway Inhibition
Source: FASEB J. 2026 Aug 28;40(17):e72252. doi: 10.1096/fj.202601110R (PMC13524200; doi:10.1096/fj.202601110R)
Supplement: Supplementary file 3 [file FSB2-40-e72252-s004.pdf]

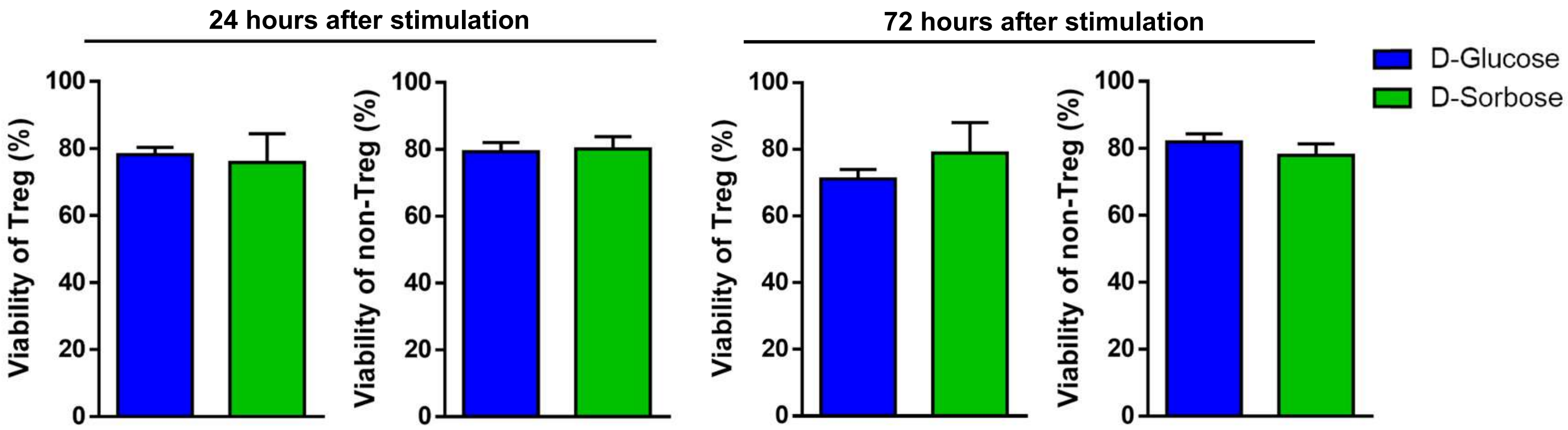

Supplemental figure 3. d-Sorbose does not affect Treg cell viability

Treg ($CD3^{+}CD4^{+}CD25^{+}$) and non-Treg ($CD3^{+}CD4^{+}CD25^{-}$) cells were isolated from the spleens of C57BL/6J mice using magnetic-activated cell sorting (MACS). Isolated cells were seeded in triplicate and cultured in medium containing 25 mM d-sorbose or d-glucose for 24 or 72 h under stimulation with anti-CD3/CD28 beads and recombinant interleukin-2 (rIL-2). After culture, the cells were collected, and cell viability was assessed by trypan blue staining. Data are presented as the mean ± standard deviation. Statistical analyses were performed using the unpaired Student's t-test.
