# Supplementary material for: d‐Sorbose Promotes Regulatory T‐Cell Differentiation and Ameliorates Autoimmune and Allergic Diseases via Hexosamine Biosynthetic Pathway Inhibition
Source: FASEB J. 2026 Aug 28;40(17):e72252. doi: 10.1096/fj.202601110R (PMC13524200; doi:10.1096/fj.202601110R)
Supplement: Supplementary file 4 [file FSB2-40-e72252-s005.pdf]

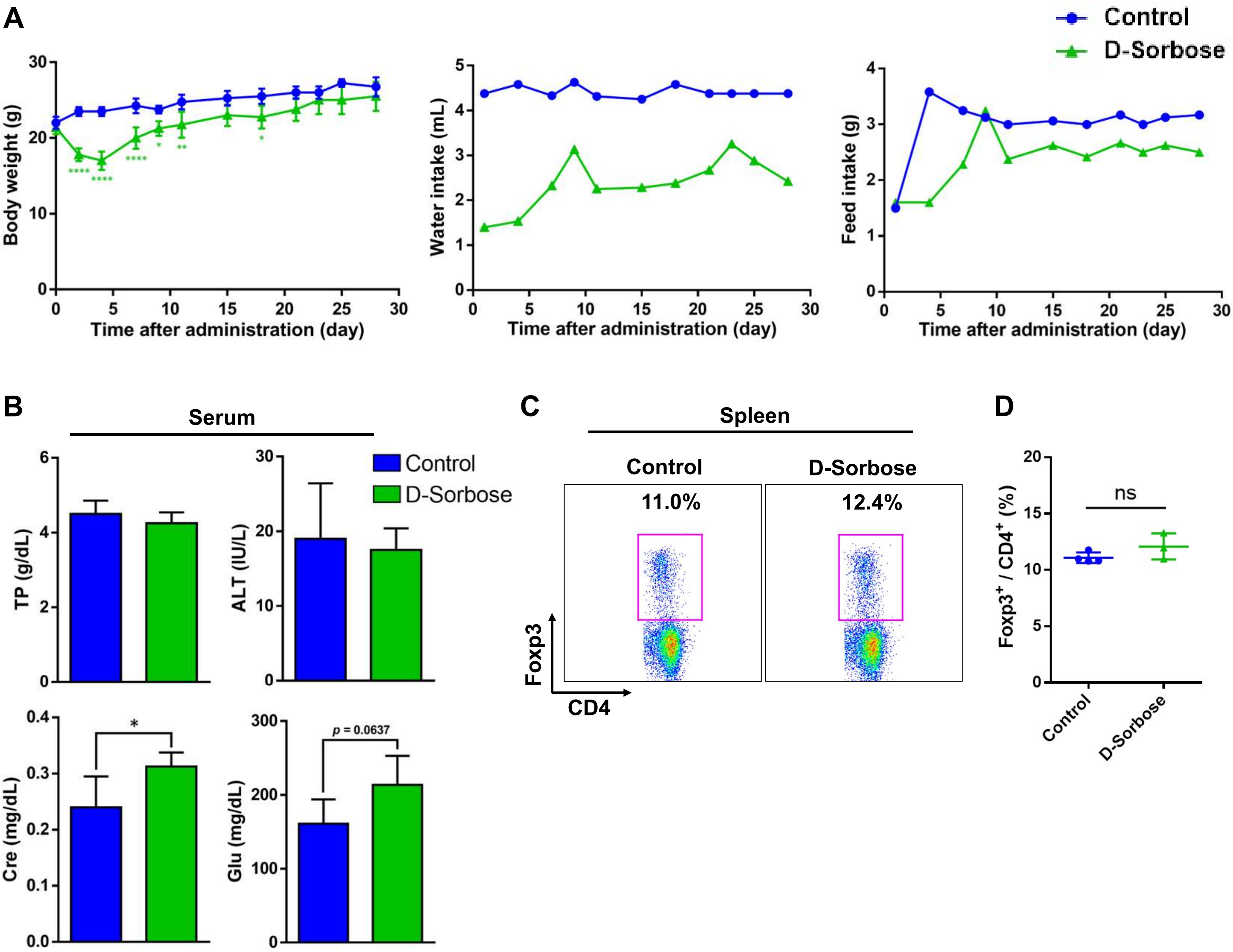

Supplemental figure 2. Safety evaluation of mice treated with 1.1 M d-Sorbose.

(a–d) C57BL/6J mice were given 1.1 M d-sorbose solution or distilled water (control) ad libitum, and serum and spleen samples were collected after 4 weeks of treatment. (a) Body weight and water and food intake during the treatment period. Water and food intake are presented as the average per cage. (b) Serum biochemical parameters. (c, d) Representative dot plots (c) and a graphical summary of the cumulative data (d) showing Treg frequency in the spleen 4 weeks after treatment. Abbreviations: TP, total protein; ALT, alanine aminotransferase; Cre, creatinine; Glu, glucose. Data are presented as the mean ± SD (n = 3–6). Statistical analyses were performed using two-way analysis of variance (ANOVA) followed by Sidak's multiple-comparison test (a) and the unpaired Student's t-test (b, d). $p < 0.05$, $p < 0.01$, $p < 0.0001$ vs. control.
