# Supplementary material for: d‐Sorbose Promotes Regulatory T‐Cell Differentiation and Ameliorates Autoimmune and Allergic Diseases via Hexosamine Biosynthetic Pathway Inhibition
Source: FASEB J. 2026 Aug 28;40(17):e72252. doi: 10.1096/fj.202601110R (PMC13524200; doi:10.1096/fj.202601110R)
Supplement: Supplementary file 5 [file FSB2-40-e72252-s001.docx]

**Supplemental Table 1. Characteristics of the monosaccharides used in the experiment.**

| **Monosaccharide** | **Category** | **M.W.** |
| --- | --- | --- |
| **D-Glucose** | **Aldohexose** | **180.16** |
| **D-Mannose** | **Aldohexose** | **180.16** |
| **D-Allose** | **Aldohexose** | **180.16** |
| **D-Psicose** | **Ketohexose** | **180.16** |
| **D-Tagatose** | **Ketohexose** | **180.16** |
| **D-Sorbose** | **Ketohexose** | **180.16** |
